# Molecular xenomonitoring as an indicator of microfilaraemia prevalence for lymphatic filariasis in Samoa in 2019

**DOI:** 10.1186/s13071-024-06463-7

**Published:** 2024-09-09

**Authors:** Maddison Howlett, Helen J. Mayfield, Brady McPherson, Lisa Rigby, Robert Thomsen, Steven A. Williams, Nils Pilotte, Shannon M. Hedtke, Patricia M. Graves, Therese Kearns, Take Naseri, Sarah Sheridan, Angus McLure, Colleen L. Lau

**Affiliations:** 1 School of Public Health, Faculty of Medicine, The University of Queensland, Brisbane, QLD 4006 Australia; 2 Centre for Clinical Research, The University of Queensland, Brisbane, QLD 4006 Australia; 3 Australian Defence Force Malaria and Infectious Disease Institute, Enoggera, 4051 Australia; 4 Samoa Ministry of Health, Apia, WS 1330 Samoa; 5 Department of Biological Sciences, Smith College, Northampton, MA 01063 USA; 6 Department of Biological Sciences, Quinnipiac University, Hamden, CT 06518 USA; 7 Department of Environment and Genetics, La Trobe University, Bundoora, VIC 3086 Australia; 8 College of Public Health, Medical and Veterinary Sciences, James Cook University, Cairns, 4878 Australia; 9 Menzies School of Health Research, Charles Darwin University, Casuarina, NT 0810 Australia; 10 National Centre for Immunisation Research and Surveillance, Westmead, Sydney, Australia; 11 National Centre for Epidemiology and Population Health, Australian National University, Canberra, Australia

**Keywords:** Vector-borne disease, Surveillance, Lymphatic filariasis elimination

## Abstract

**Background:**

Lymphatic filariasis (LF) is a globally significant, vector-borne, neglected tropical disease that can result in severe morbidity and disability. As the World Health Organization (WHO) Global Programme to Eliminate Lymphatic Filariasis makes progress towards LF elimination, there is greater need to develop sensitive strategies for post-intervention surveillance. Molecular xenomonitoring (MX), the detection of pathogen DNA in vectors, may provide a sensitive complement to traditional human-based surveillance techniques, including detection of circulating filarial antigen and microfilaraemia (Mf). This study aims to explore the relationship between human Mf prevalence and the prevalence of polymerase chain reaction (PCR)-positive mosquitoes using MX.

**Methods:**

This study compared Mf and MX results from a 2019 community-based survey conducted in 35 primary sampling units (PSUs) in Samoa. This study also investigated concordance between presence and absence of PCR-positive mosquitoes and Mf-positive participants at the PSU level, and calculated sensitivity and negative predictive values for each indicator using presence of any Mf-positive infection in humans or PCR-positive mosquitoes as a reference. Correlation between prevalence of filarial DNA in mosquitoes and Mf in humans was estimated at the PSU and household/trap level using mixed-effect Bayesian multilevel regression analysis.

**Results:**

Mf-positive individuals were identified in less than half of PSUs in which PCR-positive mosquito pools were present (13 of 28 PSUs). Prevalence of PCR-positive mosquitoes (each species separately) was positively correlated with Mf prevalence in humans at the PSU level. Analysed at the species level, only *Aedes polynesiensis* demonstrated strong evidence of positive correlation (*r*) with human Mf prevalence at both PSU (*r*: 0.5, 95% CrI 0.1–0.8) and trap/household levels (*r*: 0.6, 95% CrI 0.2–0.9).

**Conclusions:**

Findings from this study demonstrate that MX can be a sensitive surveillance method for identifying residual infection in low Mf prevalence settings. MX identified more locations with signals of transmission than Mf-testing. Strong correlation between estimated PCR-positive mosquitoes in the primary vector species and Mf in humans at small spatial scales demonstrates the utility of MX as an indicator for LF prevalence in Samoa and similar settings. Further investigation is needed to develop MX guidelines to strengthen the ability of MX to inform operational decisions.

**Graphical abstract:**

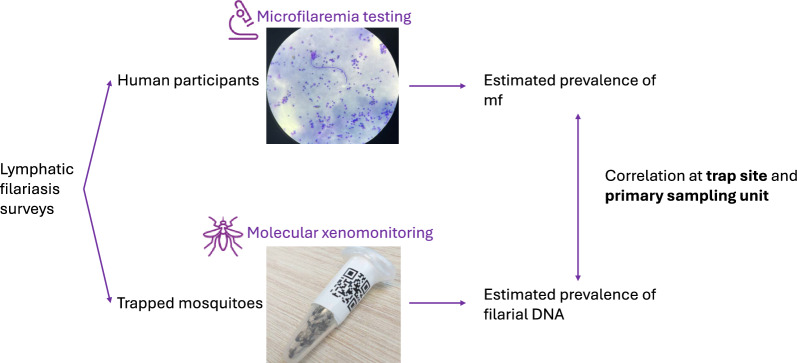

**Supplementary Information:**

The online version contains supplementary material available at 10.1186/s13071-024-06463-7.

## Background

Lymphatic filariasis (LF) is a mosquito-borne disease that can result in severe morbidity and disability such as elephantiasis, lymphoedema and scrotal hydroceles [1]. As of 2021, the World Health Organization (WHO) estimated that 882 million people in 44 countries remain at risk of LF, requiring anti-filarial medication to interrupt transmission [2]. LF elimination efforts are coordinated by the WHO’s Global Programme to Eliminate Lymphatic Filariasis (GPELF), which is founded on a two-fold strategy of interrupting LF transmission and controlling LF-related morbidity [3]. The first strategy is primarily achieved through the administration of mass drug administration (MDA), which involves the distribution of anti-filarial medication to eligible populations in endemic areas, supported by surveillance activities. Effective and sensitive surveillance is essential for successful elimination, as it indicates when intervention is needed or when prevalence is below a threshold where it is presumed that transmission cannot be sustained. While significant progress has been made by the GPELF, ongoing endemicity in a number of regions [4] highlights the need for high-quality surveillance to further support elimination efforts.

Traditional surveillance programmes use human-based indicators, including antigen (Ag) and microfilaraemia (Mf) [5]. While Ag testing is rapid and convenient in a field setting [6], it does not differentiate between an active infection or an infection that has been recently cleared [7]. Conversely, Mf testing captures active infection but can be resource and labour intensive, particularly in regions requiring night surveys due to nocturnal Mf periodicity (Mf circulating in the peripheral blood during a few hours around midnight) [8]. In addition, Mf may not be present in infected persons if adult worms are too young, too old or have not mated, and detection may be missed in cases where parasite density is low [9].

Molecular xenomonitoring (MX), a vector-based tool, may provide a sensitive complement to the traditional surveillance strategies. MX involves testing mosquitoes for filarial DNA using polymerase chain reaction (PCR) and is underpinned by the assumption that an infected mosquito can be used as a proxy measure for an infectious human nearby [9]. Promising results for the use of MX in field settings have been reported in American Samoa, India and Sri Lanka, among others [10–14]. Despite this, there are no formal guidelines for the use of MX in LF surveillance efforts. This is likely due to insufficient evidence on how mosquito infection prevalence relates to human infection prevalence [11].

Previous work evaluating MX for LF surveillance has focussed on Ag as an indicator of human infection. While comparative MX and Ag analyses have demonstrated associations between the two indicators across a number of survey locations [9, 15, 16], the inability of Ag to distinguish between an active infection and a recently cleared infection is a challenge when interpreting results from post-intervention settings. Results from 2018 and 2019 community surveys conducted in Samoa noted a significant decline in estimated prevalence of PCR-positive mosquitoes 10–12 months after the first round of triple-drug MDA (diethylcarbamazine, albendazole and ivermectin), but no corresponding change in Ag prevalence 8–10 months post-MDA [9]. As Mf testing represents active infection, it may provide a more comparable indicator for MX validation as a surveillance tool in post-intervention settings. This study aimed to explore the relationship between the prevalence of Mf-positive humans and the prevalence of PCR-positive mosquitoes using MX.

The objectives of this study were to (i) investigate whether LF transmission is still occurring in Samoa post-MDA, (ii) compare the presence and concordance of PCR-positive mosquito pools and Mf-positive humans at the PSU level and (iii) investigate the relationship between estimated prevalence of PCR-positive mosquitoes and prevalence of Mf-positive humans at PSU and household/trapping site levels.

## Methods

### Data sources

#### Study setting

Samoa is located in the South Pacific, and consists of two main islands, Upolu and Savai’i, and six islets. *Wuchereria bancrofti* is the only known species of filarial worm in Samoa and has been endemic in Samoa since at least the 1920s, with a recorded Mf prevalence of 19.1% prior to the first attempt to control transmission [17]. The primary vector for LF in Samoa is the day-biting *Aedes polynesiensis,* with other *Aedes* species also contributing to transmission [18, 19]. Since joining the Pacific Programme to Eliminate LF (PacELF) in 1999, Samoa has conducted eight nationwide rounds of two-drug MDA (diethylcarbamazine and albendazole) and two targeted rounds in 2015 and 2017 [19, 20]. After failing Transmission Assessment Surveys (TAS) for LF in part of the country in 2013 and nationwide in 2017, Samoa became the first country to distribute a nationwide triple-drug MDA (diethylcarbamazine, albendazole and ivermectin) in August 2018 [21].

Samoa’s population was an estimated 200,874 people in 2019, with the majority of residents living in rural areas [22]. The country is divided into four administrative regions, with approximately 20% of the population residing in Apia Urban Area (AUA), 33% in North-West Upolu (NWU), 24% in Rest of Upolu (ROU) and 24% in Savai’i (SAV) [22]. Samoa has a tropical climate, with average rainfall between 3000 and 6000 mm/year and largely forested inland areas [23].

#### Selection of primary sampling units (PSUs) and households

Both the human and mosquito survey used a community-based, cross-sectional, cluster design. Data were collected in 35 primary sampling units (PSUs) located across Upolu, Savai’i and Manono islands. Of the 35 PSUs, 30 were randomly selected using methodology previously published [9, 24]. The remaining five villages were purposively selected by the Samoa Ministry of Health due to high Ag prevalence in previous surveys. This study used data from both randomly and purposively selected PSUs.

Within each PSU, 15 households were selected using a ‘virtual walk’ method [24]. If the selected location was not an inhabited household, it was replaced with the closest house. Where PSUs contained more than one village, the number of houses selected in each village was proportional to village population. Country, region and village boundaries were obtained from the Pacific Data Hub and DIVA-GIS, and geographic information systems software ArcMap was used to manage spatial data [9].

#### Mf testing

The Ag and Mf survey in humans was conducted between 28 March and 17 May 2019 (6–8 months post-MDA). The survey comprised two components: a household survey (age ≥ 5 years) and a convenience survey aimed at recruiting children aged 5–9 years. To ensure the human and MX surveys were comparable in design and effort, this analysis includes only human data from the household survey component. As previously described [24], the Alere Filariasis Test Strip (FTS) was used to detect Ag on each blood sample, and where sufficient blood was available, Ag-positive samples were retested to confirm the result. For all Ag-positive samples, up to three thick blood smear slides were prepared according to WHO guidelines [25]. Slides were examined independently by two trained parasitologists in Australia, and a participant was classified as Mf-positive if Mf were observed on one or more slides. As readers examined different slides for each Ag-positive sample, there was potential for variance in observations. The level of agreement between the two readers was calculated by assessing concordance of presence and absence results, and Mf densities were calculated by averaging counts recorded by the two readers.

#### Mosquito survey

The mosquito survey was conducted between 20 May and 6 July 2019 (9–10 months post-MDA), following previously described methods [9]. Trap sites were located outside selected houses, at the same houses as the human survey if possible. In cases where traps could not be placed at a previously surveyed household, alternative locations were identified as close as possible with guidance from the local field team. Household coordinates were collected for all trap locations. BioGents Sentinel Mosquito Traps (models 1 and 2 with BG-Lure cartridges as attractants – BioGents, Regensburg, Germany) were used. Traps were left on-site for 48 h, and serviced once daily for mosquito bag collection and battery replacement. Female mosquitoes were sorted into eight categories using taxonomic keys: *Ae. polynesiensis, Ae. aegypti, Ae. albopictus, Ae. upolensis, Ae. (Finlaya)* spp.*, Aedes* spp. (other)*, Cx. quinquefasciatus* and *Culex* spp. (other). Where mosquitoes could not be classified at a species level, they were identified at genus level, that is, *Aedes* spp. (other) or *Culex* spp. (other). Mosquitoes were pooled into 1–25 mosquitoes per pool, sorted on the basis of taxonomic category and trap site. Pooled mosquitoes were oven dried at 60 °C for 3 h and shipped to Smith College, USA for analysis. DNA was extracted using DNeasy Blood and Tissue Kit (Qiagen, Hilden, Germany) on the basis of previously described protocols [14]. A real-time PCR assay [26] was used to test for the presence of *W. bancrofti* in each pool.

### Data analysis

Human and mosquito data were linked by pairing mosquito traps with the households sampled during the human survey on the basis of proximity. Households were paired with the nearest trap site, unless that trap site was paired with another household, in which case houses were paired with the next closest trap site. Traps and households were only paired if they were within 100 m of each other [8], which broadly corresponds to the flight range of *Ae. polynesiensis* [27]. Where traps and surveyed houses were unable to be paired, records were excluded from the Bayesian multilevel modelling but included in other analyses at the PSU level. Statistical analysis was conducted using R software (R Core Team, R version 4.3.1, Vienna, Austria) [28, 29].

#### Microfilariae prevalence in humans

The *getPrevalence* function in PoolTestR was used to estimate Mf prevalence in humans at the national and PSU levels from the Bayesian models created.

#### Prevalence of PCR-positive mosquitoes

Prevalence of mosquitoes infected with *W. bancrofti* was estimated using the PoolTestR package [30]. National estimates were calculated using the *HierPoolPrev* function to account for clustering at the PSU and trap levels. PSU level estimates were calculated using the *getPrevalence* function in PoolTestR from regression modelling. Due to insufficient catch numbers, prevalence estimates for *Culex* spp. (other) are not reported in this paper.

#### Infection presence and absence, by PSU

Mf presence in humans was defined as one or more Mf-positive samples in a PSU. Infection in mosquitoes was defined as one or more PCR-positive mosquito pool. The level of pathogen prevalence in humans and mosquitoes may impact the degree of concordance observed between infection in mosquitoes and Mf presence in humans at the PSU level. As Ag testing is the primary tool used for LF transmission assessment [31], crude Ag prevalence was used to classify villages into a ‘lower’ prevalence category (≤ 2.5%) and a ‘higher’ prevalence category (> 2.5%). This cut-off was based on the median Ag prevalence across the PSUs in 2019. Concordance between presence or absence of human and mosquito indicators was calculated at the PSU level, with agreement between households containing Mf-positive residents and matched trap locations that collected a PCR-positive mosquito pool measured using the Cohen’s kappa statistic (*κ*). A Cohen’s kappa statistic value of ≤ 0.2 was considered to indicate no or slight agreement.

#### Sensitivity and NPV by PCR-positive pools and Mf presence in humans

Sensitivity and negative predictive values (NPV) were calculated for Mf-positive humans and PCR-positive mosquito pools at the PSU level. For this analysis, the presence of infection in a PSU was defined as any positive indicator detected in the PSU, that is, either a PCR-positive mosquito pool and/or a Mf-positive person. As the presence of any human or mosquito infection indicator was used as a ‘reference’ for the calculations, it was not possible to detect true false positives. Specificity and positive predictive value (PPV) were therefore not calculated.

#### Bayesian multilevel modelling

Bayesian multilevel modelling was used to quantify the correlation between pathogen markers in humans and mosquitoes at the PSU and household levels. As the sample size at each of these areas were small, this study used small area estimation (SAE) techniques to estimate the prevalence of filarial indicators in humans and mosquitoes. SAE produces more precise estimates for each small area by ‘borrowing information’ from the broader survey population [32, 33]. When there are multiple outcomes (e.g. pathogen prevalence in multiple mosquito species), SAE additionally leverages the correlation of the outcomes within small areas to improve estimates for each outcome in each cluster [34]. Bayesian multivariate logistic models that jointly predicted the prevalence of PCR-positive mosquitoes and Mf in humans at each PSU and collection site (household) were fitted using correlated (multivariate) normal random effects at the PSU and trap levels [35]. Using a multivariate model allowed us to estimate the correlation of the prevalence of filarial indicators in mosquito and humans at the PSU or trap level and use any mutual information in these correlations to improve prevalence estimates for all indicators [32, 33]. While all models included human Mf data, variants of the model were fitted to predict prevalence by mosquito species, mosquito genus, or for all mosquitoes combined. The *PoolRegBayes* function in PoolTestR [30] was used to fit the models, which allowed the models to adjust for the size of mosquito pools to estimate mosquito-level infection prevalence. Estimates of prevalence for filarial markers in mosquitoes and humans were extracted at the PSU and trap site (household) levels from the fitted models. Correlation coefficients (*r*) of the multivariate normal random effects for PSU and collection site were also extracted from fitted models.

Scatterplots were produced using the ggplot2 package [36] in R to visualise the correlation of the prevalence between these markers at the PSU level. The correlation of the prevalence between markers are reported at the trap site level, but are not visually presented in this paper. Due to the partial-pooling effect of random effect models, estimates of prevalence for each disease marker in each small area (PSU or trap/household) are pulled towards the mean values at the higher levels; that is, PSU-level estimates towards the national prevalence estimate and household/trap-level estimates towards the parent PSU estimate. Consequently, estimates of prevalence were > 0% for all measures and areas, even when all humans/pools in the PSU or trap/household site were negative for filarial mf/filarial DNA.

## Results

### Prevalence of microfilaremia in humans

The human seroprevalence survey collected samples from 2597 participants, of whom 117 were Ag-positive. Mf slides prepared from 117 participants with Ag-positive blood samples, and 31 participants (26.5% of the Ag-positive and 1.2% of total participants) from 13 PSUs were positive for Mf, with a density ranging from 16.7 to 2371.4 Mf per mL. The level of agreement of presence or absence of Mf between the two readers was 95.4%. When calculated from the Bayesian multilevel model including correlations between human and mosquito pathogens markers, the national prevalence of Mf was estimated to be 1.6% (95% CrI 0.9–2.8).

### Abundance and prevalence of PCR-positive mosquitoes

A total of 34,276 mosquitoes (2498 pools, average 13.72 per pool, range 1–25, SD 9.52) were collected. The number of mosquitoes collected per PSU ranged from 290 to 3718 (average 979 per PSU). The majority (60.8%) of pools processed were of *Aedes* genus (16,445 mosquitoes). However, among species or species groups, *Cx. quinquefasciatus* was the most common overall for both number of pools and mosquitoes (970 pools, 17,807 mosquitoes).

The highest estimated prevalence of PCR-positive mosquitoes was found among *Ae. polynesiensis* (0.4%, 95% CrI 0.1–0.8)*,* followed by *Ae. aegypti* (0.3%, 95% CrI 0.0–1.0) (Table 1).

**Table 1:** Summary of female mosquitoes caught in all primary sampling units (PSUs) in Samoa in 2019, with estimated prevalence of PCR-positive mosquitoes, stratified by mosquito genus (bold) and species

Species/Genus	Number of mosquitoes	Number of pools	Number of positive pools	Estimated prevalence of PCR-positive mosquitoes (95% CrI)
***Aedes***	**16,445**	**1520**	**144**	**0.30% (0.10–0.60)**
*Ae. polynesiensis*	10,536	692	102	0.39% (0.14–0.78)
*Ae. (Finlaya)* spp.	1377	185	2	0.07% (0.00–0.35)
*Ae. aegypti*	3396	445	33	0.27% (0.00–0.97)
*Aedes* spp. (other)	1136	198	7	0.23% (0.00–0.84)
***Culex***	**17,831**	**978**	**28**	**0.08% (0.02–0.10)**
*Cx. quinquefasciatus*	17,807	970	28	0.08% (0.02–0.16)
**Total**	**34,276**	**2498**	**172**	**0.22% (0.10–0.39)**

Results for *Culex* spp. other are not presented due to small collection numbers and unreliable estimates

### Presence and absence of infection, by PSU

Of the 35 PSUs, 13 (37%) had at least one Mf-positive person detected and 28 PSUs (80%) had one or more PCR-positive mosquito pools (Table 2). Overall, there was low concordance (40%) between the human and mosquito indicators, with both Mf-positive humans and PCR-positive mosquito pools identified in ten PSUs. In four PSUs, we did not identify any Mf-positive participants or PCR-positive mosquito pools.

**Table 2:** Comparison of the presence or absence of lymphatic filariasis infection indicators in humans (microfilaria) and mosquitoes (filarial DNA) by primary sampling units (PSUs) in Samoa, 2019

PSU	Total number of PSUs	No. (%) of PSUs with Mf-positive humans	No. (%) of PSUs with PCR-positive mosquito pools	Concordance (%)	Cohen’s kappa statistic
All	35	13 (37%)	28 (80%)	40	− 0.04
Lower Ag prevalence (≤ 2.5%)	18	3 (17%)	14 (40%)	17	−0.21
Higher Ag prevalence (> 2.5%)	17	10 (59%)	14 (40%)	65	0.20

PSUs were stratified by *W*. *bancrofti* antigen prevalence

**Fig. 1:**
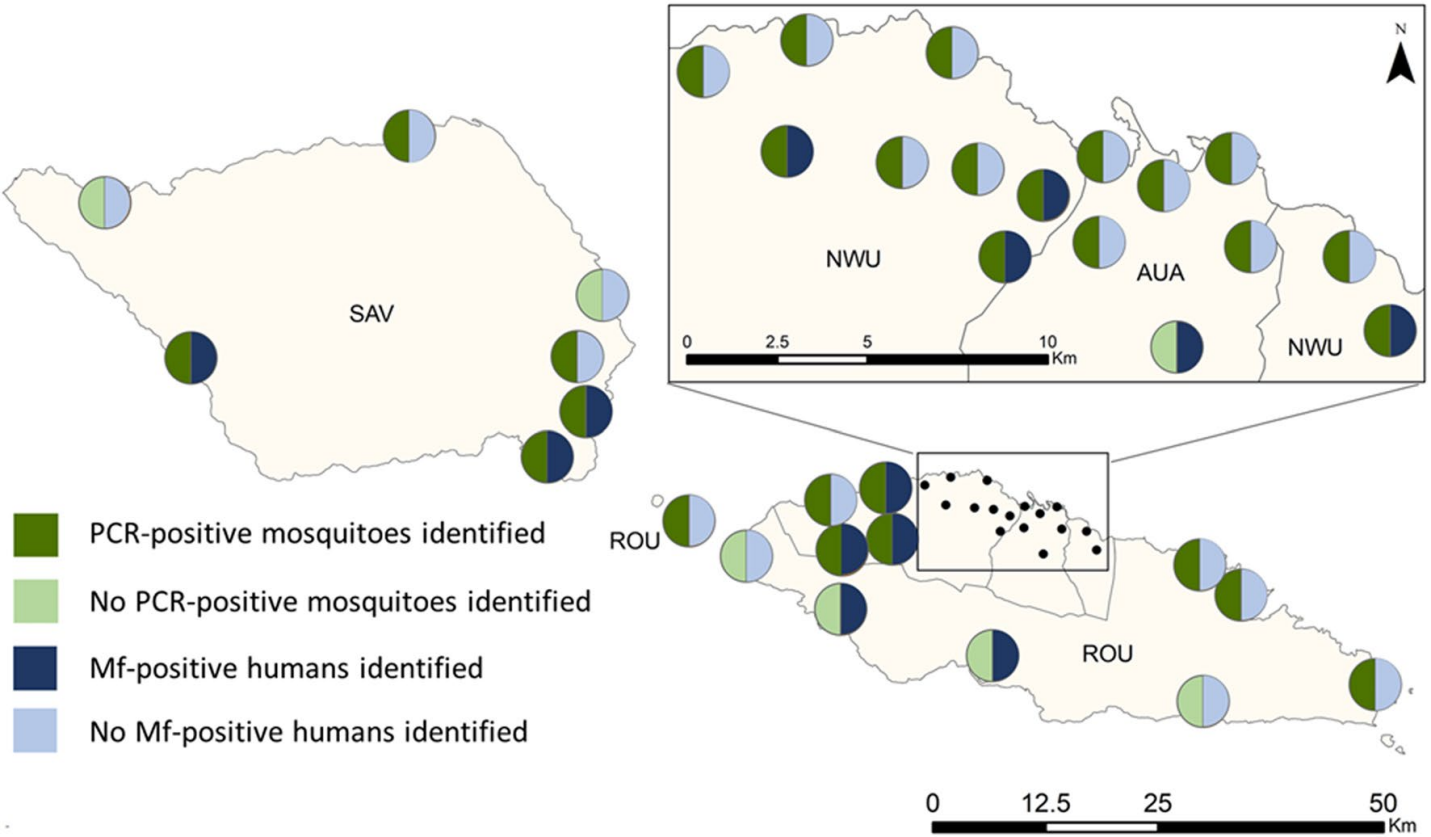
Presence or absence of microfilaria (Mf) in humans and PCR-positive mosquito pools by primary sampling units (PSUs) in Samoa, 2019. Each circle represents a PSU. Regions are *AUA* Apia Urban Area, *NWU* North-West Upolu, *ROU* Rest of Upolu, *SAV* Savai’i

When PSUs were stratified by Ag prevalence, concordance differed substantially. Lower Ag prevalence PSUs (*n* = 18) demonstrated 17% concordance between presence of Mf-positive humans and PCR-positive mosquitoes, or absence of Mf-positive humans and PCR-positive mosquitoes, compared with 65% concordance observed in higher Ag prevalence PSUs (*n* = 17). Cohen’s kappa calculations indicate no agreement (*κ* = −0.2) between the Mf and MX survey results in lower Ag prevalence PSUs compared with a low agreement (*κ* = 0.2) in higher Ag prevalence PSUs. Figure 1 compares the presence/absence of human and mosquito indicators by PSU.

Overall, presence of a PCR-positive mosquito pool had a much higher sensitivity (90.3%) than Mf presence in humans (41.9%) of detection of any Mf and/or MX indicators of infection in a PSU (Table 3). PCR-positive pools also had a higher NPV than the presence of Mf-positive humans (57.1% compared with 18.2%, respectively).

**Table 3:** Correlation coefficient (*r*) between estimated filarial DNA prevalence in female mosquitoes and microfilaria (Mf) prevalence in humans at the primary sampling unit and trap site/household levels in Samoa in 2019, by mosquito genus (bold) and species

	Correlation coefficient (95% CrI)	
Species/Genus	Primary sampling unit	Trap site/household
***Aedes***	*0.58 (0.12–0.90)	*0.74 (0.38–0.97)
*Ae. polynesiensis*	*0.46 (0.03–0.80)	*0.57 (0.16–0.87)
*Ae. (Finlaya)* spp.	0.14 (−0.44 to 0.67)	0.06 (−0.57 to 0.69)
*Ae. aegypti*	0.39 (−0.12 to 0.81)	0.41 (−0.03 to 0.77)
*Aedes* spp. (other)	0.40 (−0.13 to 0.82)	0.43 (−0.06 to 0.82)
***Culex***	**0.24 (−0.37 to 0.79)**	**−0.10 (−0.86 to 0.80)**
*Cx. quinquefasciatus*	0.14 (−0.36 to 0.63)	−0.04 (−0.66 to 0.62)

^*^Credible intervals indicate a high (> 97.5%) posterior probability of positive correlation

### Correlation between estimated prevalence of PCR-positive mosquitoes and Mf in humans by PSU and trap site (household)

**Fig. 2:**
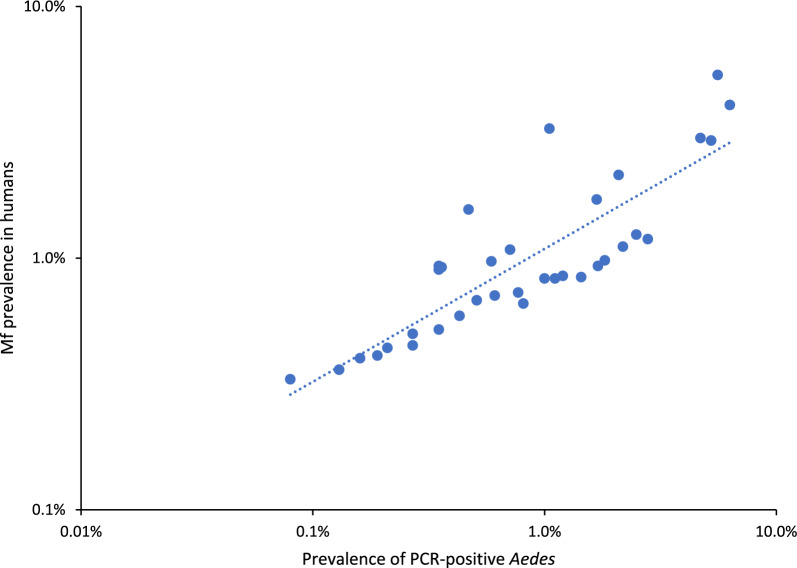
Correlation between estimated prevalence of PCR-positive mosquitoes among female *Aedes* and estimated microfilaria (Mf) prevalence in humans by primary sampling units (PSUs) in Samoa, 2019. Dots represent an individual PSU. Prevalence of PCR-positive *Aedes* mosquitoes and the prevalence of Mf in humans estimated by a Bayesian multilevel logistic regression model, adjusting for clustering at the PSU level and trap site and the number of mosquitoes in each pool. Results presented on logarithmic scales

**Fig. 3:**
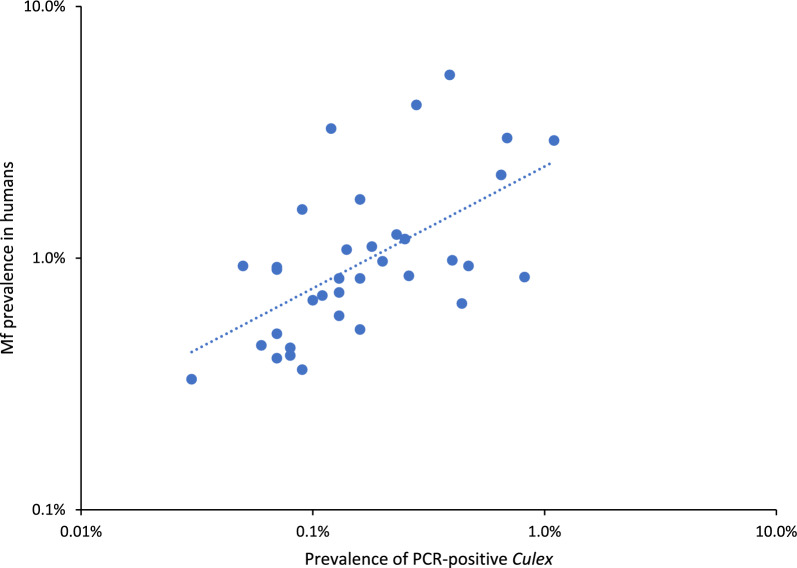
Correlation between estimated filarial DNA prevalence among female *Culex* mosquitoes and estimated microfilaria (Mf) prevalence by primary sampling units (PSUs) in Samoa, 2019. Dots represent an individual PSU, plotted against the prevalence of *W*. *bancrofti* filarial DNA among *Culex* mosquitoes and the prevalence of Mf for the PSU, as estimated by a Bayesian multilevel logistic regression model adjusting for clustering at the PSU level and trap site and the number of mosquitoes in each pool. Figure presented on logarithmic axes

At the mosquito genus category, multivariate regression modelling showed a strong, positive correlation at the PSU level between Mf prevalence in humans and prevalence of PCR-positive mosquitoes among all combined *Aedes* (0.6, 95% CrI 0.1–0.9) but not in *Culex* (Table 3, Figs. 2, 3). When assessed at the trap site level, prevalence of PCR-positive *Aedes* was even more strongly correlated with Mf prevalence in humans (0.7, 95% CrI 0.4–1.0) (Table 3). When analysed by species, significant correlation with human Mf prevalence was only present for the primary vector species, *Ae. polynesiensis* (0.5, 95% CrI 0.1–0.8). Correlation between prevalence of PCR-positive *Culex* genus and *Aedes* genus demonstrated a moderate, positive correlation at the PSU level (0.6, 95% CrI 0.1–0.9).

## Discussion

This study demonstrates the utility of MX to provide a sensitive complement to traditional surveillance of humans in low Mf prevalence settings. Compared with Mf prevalence in humans, PCR-positive mosquito pools demonstrated a higher sensitivity and NPV for identifying a signal of LF infection at the PSU level. This study also found a positive correlation between estimated Mf prevalence in humans and estimated filarial DNA prevalence in *Aedes* mosquitoes, the primary vector genus in Samoa, at both the household-trap site and PSU levels. This relationship between Mf and MX survey results aligns with findings from a 2021 meta-analysis, which demonstrated a strong linear relationship between the prevalence of PCR-positive wild-caught mosquitoes and Mf prevalence across 24 studies on LF-endemic areas around the globe [37]. The results presented here, particularly when combined with a previous study in Samoa reporting significant associations between filarial DNA prevalence in mosquitoes and Ag prevalence [9], provide strong support for the value of MX in the Pacific Islands.

The MX survey yielded a much higher power for LF signal detection at the PSU level compared with Mf in humans on the basis of a similar survey effort. While both surveys utilised the same number of collection sites and a similar survey time in terms of fieldwork hours, the sample sizes for each survey differed greatly (2597 human participants compared with 32,765 mosquitoes tested in 2498 pools). From an operational perspective, this translated to a higher sensitivity and NPV for PCR-positive pools compared with Mf-positive humans. As there is no ‘gold standard’ method for LF detection in a large-scale field setting, sensitivity was based on a positive infection indicator that captured any detection of an infection indicator in a PSU, including a PCR-positive pool and/or a Mf-positive person. Therefore, the disparity in sensitivity values can be attributed to the higher prevalence of PCR-positive pools compared with Mf-positive individuals. While no formal cost–benefit analysis was conducted, the higher prevalence of PCR-positive pools across both purposively and randomly selected PSUs demonstrates the potential cost-effectiveness of MX in low-prevalence settings for detecting LF infection. This adds to findings from McPherson et al. which suggest that in a post-MDA setting, MX may be more sensitive than Ag testing for detecting changes in transmission [9].

This study explored both mosquito species- and genus-level correlations between the prevalence of Mf-positive humans and prevalence of PCR-positive mosquitoes by household-trap site and PSU. Genus-level analysis yielded stronger correlation than species-level analysis, likely due to the larger sample sizes when aggregating across species. However, correlations were still evident among the species-level analyses. This study found a moderate, positive correlation between filarial DNA infection prevalence among *Ae. polynesiensis*, the primary vector species in Samoa, and Mf prevalence in humans at the PSU level. At the trap site level, the strength of this correlation was even higher. No such correlation was found for other mosquito species, including *Cx. quinquefasciatus*, which was the most abundant species collected throughout the survey. A possible explanation for these different results is the flight ranges of species. The flight range for *Aedes* genus mosquitoes is estimated to be quite short, at 50–200 m from their breeding containers [38]. In comparison, the *Culex* genus have an estimated flight range of around 3 km from their breeding sites [39]. As analysis was conducted at the PSU and household levels, it is possible that a higher dispersion of *Culex* mosquitoes led to the capture of infected mosquitoes outside of the PSUs where they had taken blood meals. This hypothesis could be further investigated with geospatial modelling to explore correlations between human and mosquito indicators over different distances.

For both *Aedes* and *Culex*, the comparison between species- and genus- level correlation values suggest that in the Samoan LF context, a genus-based approach may be more practical for calculating MX-based thresholds than a species-based specification. There are operational advantages to this approach, as processing mosquitoes on the basis of genus-level pools removes the need to sort mosquitoes into species, which can be time-consuming and require specialist expertise. This is further supported by results published by McPherson et al. [9], which found that sorting mosquitoes by genus or species level made little difference to prevalence results for the purposes of detecting temporal changes.

The primary challenge of this study was the difficulty in identifying correlations between pathogen markers in human and mosquito populations at small areas (PSUs and households) when the prevalence of all pathogen markers were low. This is a commonly cited challenge among studies that compare surveillance tools against Mf prevalence as a reference standard [37], and can often impact calculated sensitivity of comparative tools. The low overall prevalence of pathogen markers in humans and mosquitoes combined with the reduced sample sizes at the PSU level and household levels limited the power of detection at any given small area. This limitation was highlighted by the presence/absence analysis, which showed limited concordance between Mf positive humans and PCR-positive mosquito pools in PSUs with high antigen prevalence, and no concordance in PSUs with low antigen prevalence. Despite this, our study yielded operationally useful insights from sparse data using small area estimation methods to overcome this limitation [35]. Use of small area estimation allowed us to confirm correlation in prevalence between mosquitoes and Mf in humans, where the use of standard regression and descriptive statistics in other studies have often failed to discern a relationship [37]. Though not the focus of this study, these correlations could also be used to improve the estimates of infection prevalence where mosquitoes were sampled but humans were not, or to improve the estimates of Mf prevalence in locations where both human and MX surveillance were undertaken but the number of humans sampled was small [19].

This study found MX to be more sensitive than Mf surveillance in humans at identifying locations with *W. bancrofti* infections for a comparable survey effort. As MX may provide a highly effective complementary or alternative surveillance method that overcomes challenges associated with human surveillance in some settings, determining how MX can be used to inform operational decisions is critical. This study demonstrates that the prevalence of pathogen markers in vectors is correlated to human infection markers at small (household) and medium (PSU) spatial scales, and illustrates the use of small area estimation techniques to utilise these observed correlations to improve prevalence estimates. In regions where MX is a viable tool for LF surveillance, comparative studies should be incorporated into work programmes for better understanding of prevalence correlation across a range of regions.

## Supplementary Information

Additional file 1.

## Data Availability

All relevant data are within the paper. We are unable to provide individual-level antigen prevalence data and demographic data because of the potential for breaching participant confidentiality. The communities in Samoa are very small, and individual-level data such as age, sex and village of residence could potentially be used to identify specific persons. For researchers who meet the criteria for access to confidential data, the data are available on request from the Human Ethics Officer at the Australian National University Human Research Ethics Committee, email: human.ethics.officer@anu.edu.au.
