# Supplementary material for: Molecular xenomonitoring as an indicator of microfilaraemia prevalence for lymphatic filariasis in Samoa in 2019
Source: Parasit Vectors. 2024 Sep 9;17:382. doi: 10.1186/s13071-024-06463-7 (PMC11386098; doi:10.1186/s13071-024-06463-7)
Supplement: Supplementary file 1 — Additional file 1. [file 13071_2024_6463_MOESM1_ESM.pdf]

Molecular xenomonitoring as an indicator of microfilaraemia prevalence for lymphatic filariasis in Samoa in 2019: Supplementary material

Authors: Maddison Howlett, Helen J Mayfield, Brady McPherson, Lisa Rigby, Robert Thomsen, Steven A Williams, Nils Pilotte, Shannon Hedtke, Patricia M. Graves, Therese Kearns, Take Naseri, Sarah Sheridan, Angus McLure*, Colleen L Lau*

*Contributed equally

Table 1 Prevalence (%) of filarial DNA in mosquitoes (*Aedes* and *Culex*) and microfilaraemia (Mf) prevalence (%) in humans in Samoa 2019. Prevalence estimated using multilevel Bayesian model, stratified by region and primary sampling unit (PSU).

PSUs by region	Prevalence of filarial DNA in *Aedes* (95% CrI)	Prevalence of filarial DNA in *Culex* (95% CrI)	Prevalence of *Mf* in humans (95% CrI)
Apia Urban Area (AUA)			
Avele + Letava	0.35 (0.02–1.38)	0.07 (0.00–0.27)	0.90 (0.08–3.27)
Fugalei + Vaimea	0.27 (0.01–1.09)	0.07 (0.00–0.28)	0.50 (0.01–1.94)
Vaiala Tai + Vaiala Uta	0.61 (0.12–1.68)	0.11 (0.00–0.40)	0.71 (0.04–2.43)
Vaimoso	1.82 (0.38–4.90)	0.4 (0.09–1.14)	0.98 (0.05–3.14)
Vaitoloa	1.00 (0.15–2.93)	0.13 (0.00–0.48)	0.83 (0.05–2.81)
Vaivase Tai	0.51 (0.08–1.47)	0.10 (0.00–0.40)	0.68 (0.03–2.39)
North-West Upolu (NWU)			
Faleasiu	6.31 (2.73–12.13)	0.28 (0.01–1.00)	4.06 (1.13–9.62)
Fasitoo Tai	4.71 (1.99–9.21)	0.69 (0.12–2.00)	3.00 (0.69–7.80)
Fasitoouta	2.09 (0.47–5.26)	0.65 (0.10–2.28)	2.14 (0.37–5.99)
Laulii	5.59 (2.24–10.89)	0.39 (0.04–1.23)	5.34 (1.58–12.72)
Leauvaa	2.49 (0.69–5.94)	0.23 (0.01–0.84)	1.24 (0.1–3.88)
Letogo	0.35 (0.05–1.07)	0.16 (0.02–0.46)	0.52 (0.02–1.88)
Lotosoa	5.24 (2.44–10.06)	1.10 (0.22–3.28)	2.93 (0.54–8.07)
Nuu	2.18 (0.57–5.17)	0.18 (0.02–0.51)	1.11 (0.09–3.41)
Puipaa	0.81 (0.03–3.24)	0.44 (0.05–1.56)	0.66 (0.02–2.43)
Tuanai	0.77 (0.11–2.31)	0.13 (0.00–0.51)	0.73 (0.04–2.45)
Ululoloa	0.59 (0.04–2.08)	0.20 (0.03–0.61)	0.97 (0.09–3.24)
Vailuutai	2.79 (0.79–6.66)	0.25 (0.01–0.93)	1.19 (0.08–3.6)
Vaitele Fou	1.20 (0.26–3.22)	0.26 (0.02–0.86)	0.85 (0.06–2.71)
Vaiusu	1.68 (0.16–5.86)	0.16 (0.00–0.63)	1.71 (0.30–4.81)
Rest of Upolu (ROU)			
Faleapuna + Musumusu	1.44 (0.24–4.18)	0.82 (0.14–2.56)	0.84 (0.03–2.99)
Falefa	1.70 (0.27–4.82)	0.47 (0.07–1.50)	0.93 (0.05–3.12)
Faleseela	0.35 (0.02–1.43)	0.05 (0.00–0.18)	0.93 (0.08–3.28)
Fusi	0.36 (0.02–1.35)	0.07 (0.00–0.30)	0.92 (0.09–3.26)
Manono Uta	0.27 (0.01–1.20)	0.06 (0.00–0.23)	0.45 (0.01–1.68)
Mutiatele + Saleaaumua	0.13 (0.01–0.44)	0.09 (0.00–0.32)	0.36 (0.01–1.44)
Salani + Utulaelae	0.08 (0.00–0.32)	0.03 (0.00–0.12)	0.33 (0.01–1.45)
Salua (Manono Island)	0.43 (0.06–1.31)	0.13 (0.00–0.58)	0.59 (0.03–2.02)
Savai'i			
Lalomalava + Safua	0.21 (0.03–0.68)	0.08 (0.00–0.33)	0.44 (0.01–1.67)
Lano	0.16 (0.01–0.65)	0.07 (0.00–0.30)	0.40 (0.01–1.64)
Papa + Tafua	1.05 (0.35–2.36)	0.12 (0.00–0.48)	3.27 (0.81–8.72)
Safotu	1.11 (0.25–2.77)	0.16 (0.00–0.65)	0.83 (0.05–2.67)
Sagone	0.71 (0.16–1.95)	0.14 (0.00–0.62)	1.08 (0.13–3.47)
Salelolonga	0.47 (0.06–1.53)	0.09 (0.00–0.35)	1.56 (0.24–5.11)
Sataua	0.19 (0.01–0.74)	0.08 (0.00–0.35)	0.41 (0.01–1.64)

Note: The multilevel Bayesian model included correlated random effects for the prevalence of disease markers in humans and each mosquito genus by PSU and trap. Due to the partial-pooling effect of random effect models,

estimates of prevalence for each PSU and disease marker are pulled towards the grand mean values (i.e. national prevalence for each marker). Consequently, estimates of prevalence were somewhat >0% for all measures and PSUs, even when all humans/pools in the PSU were negative for filarial mf/filarial DNA.
